# MKRN3 Interacts With Several Proteins Implicated in Puberty Timing but Does Not Influence *GNRH1* Expression

**DOI:** 10.3389/fendo.2019.00048

**Published:** 2019-02-08

**Authors:** Venkatram Yellapragada, Xiaonan Liu, Carina Lund, Johanna Känsäkoski, Kristiina Pulli, Sanna Vuoristo, Karolina Lundin, Timo Tuuri, Markku Varjosalo, Taneli Raivio

**Affiliations:** ^1^Stem Cells and Metabolism Research Program, Faculty of Medicine, University of Helsinki, Helsinki, Finland; ^2^Department of Physiology, Faculty of Medicine, University of Helsinki, Helsinki, Finland; ^3^Molecular Systems Biology Research Group, Institute of Biotechnology & HiLIFE, University of Helsinki, Helsinki, Finland; ^4^Proteomics Unit, Institute of Biotechnology, University of Helsinki, Helsinki, Finland; ^5^Department of Obstetrics and Gynecology, Helsinki University Hospital, HUH, Helsinki, Finland; ^6^New Children's Hospital, Pediatric Research Center, Helsinki University Hospital, HUH, Helsinki, Finland

**Keywords:** MKRN3, puberty timing, protein interaction, *GNRH1*, CRISPR/Cas9

## Abstract

Paternally-inherited loss-of-function mutations in makorin ring finger protein 3 gene (*MKRN3*) underlie central precocious puberty. To investigate the puberty-related mechanism(s) of *MKRN3* in humans, we generated two distinct bi-allelic *MKRN3* knock-out human pluripotent stem cell lines, Del 1 and Del 2, and differentiated them into *GNRH1*-expressing neurons. Both Del 1 and Del 2 clones could be differentiated into neuronal progenitors and *GNRH1*-expressing neurons, however, the relative expression of *GNRH1* did not differ from wild type cells (*P* = NS). Subsequently, we investigated stable and dynamic protein-protein interaction (PPI) partners of MKRN3 by stably expressing it in HEK cells followed by mass spectrometry analyses. We found 81 high-confidence novel protein interaction partners, which are implicated in cellular processes such as insulin signaling, RNA metabolism and cell-cell adhesion. Of the identified interactors, 20 have been previously implicated in puberty timing. In conclusion, our stem cell model for generation of *GNRH1*-expressing neurons did not offer mechanistic insight for the role of MKRN3 in puberty initiation. The PPI data, however, indicate that MKRN3 may regulate puberty by interacting with other puberty-related proteins. Further studies are required to elucidate the possible mechanisms and outcomes of these interactions.

## Introduction

The onset of puberty is dependent on the reactivation of hypothalamic GnRH secretion, which leads to increased gonadotropin and sex steroid secretion, secondary sex characteristics and the attainment of adult height and fertility (1, 2). In patients with central precocious puberty (CPP), puberty starts before the age of 8 years in girls (Tanner breast stage 2) and 9 years in boys (Tanner genital stage G2) (1, 3, 4). Premature central activation of GnRH secretion may be pathological due to intracranial lesions, or idiopathic when no underlying cause can be identified (4). In girls, the annual incidence of CPP varied from 15 to 29 per 1,00,000 (5), and the overall risk and incidence is higher in girls than in boys (2, 3, 6).

The genetic etiology of idiopathic CPP is not completely understood, but the clinically most relevant gene underlying CPP is makorin ring finger protein 3 gene (*MKRN3)* (7–11). *MKRN3* is a single-exon, maternally imprinted gene, which is expressed only from the paternal allele (12). Consequently, only paternally-inherited loss-of-function mutations in *MKRN3* cause CPP (7, 11). *MKRN3* belongs to the makorin family of ubiquitin ligases together with *MKRN1* and *MKRN2*. *MKRN3* is a close relative of *MKRN1*, being an intronless retrocopy of the latter (12, 13). Similar to other makorin family members, MKRN3 too consists of three zinc finger domains (C3H), one zinc RING finger domain (C3HC4) and one MKRN3-specific Cys-His domain (CH) and is expressed ubiquitously in human fetal and adult tissues (13, 14). Based on its structure, MKRN3 is predicted to function as a putative E3-ubiquitin ligase and it potentially affects gene expression, targeted protein degradation and protein function modulation via its E3 ligase activity (12–15). Interestingly, *MKRN3* is expressed in the mouse and human hypothalamus (7, 8), but the mechanistic processes by which a paternally inherited loss-of-function *MKRN3* mutation causes CPP are currently unclear.

Human pluripotent stem cells (hPSCs) have the indefinite capability of self-renewal and they can be differentiated into specialized cell types (16). To this end, we have recently described a protocol for the differentiation of *GNRH1*-expressing neurons from hPSCs (17), which offers a possibility to investigate the role of genetic factors in the regulation of *GNRH1* in humans. Clustered Regularly Inter Spaced Palindromic Repeats (CRISPR) and CRISPR-associated protein 9 (CRISPR/Cas9) has become a prevailing technology in the area of gene editing (18). In brief, the CRISPR/Cas9 approach comprises of a short guide RNA (crRNA) fused to bacterial-specific trans-activating crRNA called tracrRNA which processes the crRNA, the fusion of crRNA and tracrRNA forms tracr:cr RNA complex which directs the Cas9 enzyme to a specific locus on DNA, generating double strand breaks (18). These double strand breaks are naturally repaired by an error-prone non-homologous end joining in the absence of a donor template (19). We utilized this technique to generate bi-allelic *MKRN3* deletions in hPSCs and differentiated the knock-out (KO) cell lines into *GNRH1*-expressing neurons by employing our recently described protocol comprising dual SMAD inhibition, FGF8 treatment and Notch inhibition (17).

Most cellular proteins do not function in isolation, but perform their biological functions by interacting with each other (20). Protein-protein interactions (PPIs) form a network that operates in a coordinative manner to bring up physiological activities within a living cell (21). Mass spectrometry (MS) has evolved into an efficient technique in analyzing and characterizing PPIs (20, 22). To further investigate the putative mechanisms by which MKRN3 may affect the timing of puberty, we investigated MKRN3 PPI partners in HEK cells by employing our recently described MS-based approach, which allows the detection of stable and transient interaction partners for the protein of interest (20). This cell line was selected as a model, since HEK cells share certain characteristics of neurons (23), are widely available to researchers, and are frequently employed in biomedical research.

## Materials and Methods

### Human Pluripotent Stem Cells

Human pluripotent stem cell line H9 [46, XX, (Wicell)] was used in this study (24). hPSCs were maintained on Matrigel® (BD Biosciences) coated dishes with StemPro (Thermo Fisher Scientific) or Essential 8 medium (Thermo Fisher Scientific). During maintenance, the cells were split with 0.5 mM EDTA (Thermo Fisher Scientific, MA) and plated in 1:3–1:8 ratios. Medium was changed daily and before the differentiation, cells were plated on Geltrex-coated dishes (Thermo Fisher Scientific) and grown until >90% confluency.

### Guide RNA Design and Production

CRISPR guides were designed using https://benchling.com/ to target the only exon of *MKRN3*. Based on their off-target and specificity scores, two pairs of the best possible guides were selected. Guide RNA DNA templates (gRNAs) were prepared by PCR (gRNA-PCR) amplification based on a method previously published (25). In brief, gRNA templates having 19 bp overhangs on 5′ and 3′ ends were fused to U6 promoter and terminator sequences (tracr) using PCR mentioned above with Phusion polymerase (Thermo Fisher Scientific). Further information on the gRNA-PCR is given in the Supplementary Material. All the CRISPR related oligos and guide sequences have been listed in Supplementary Table 1.

### Generating CRISPR/Cas9 Based *MKRN3* KO Cell Lines

For generating *MKRN3* KO in hPSCs, two gRNAs targeting different locations of *MKRN3* and a plasmid encoding wild type (WT) *Streptococcus pyogenes* Cas9 (SpCas9, referred as Cas9 hereafter), Green fluorescent protein (GFP) and puromycin resistance gene were electroporated to two million H9 cells with the Neon transfection system according to the manufacturer's instructions (Thermo Fisher Scientific). A total of 4 μg plasmid DNA and 250 ng of each gRNA was used per electroporation, and the electroporated cells were plated on Matrigel®-coated dishes and supplemented with 10 μM ROCK inhibitor (Y-27632 2HCl, Selleckchem) to enhance survival of hPSCs by inhibiting dissociation-induced apoptosis (26). Culture medium was changed every 24 h, with transient selection of surviving clones using 0.12 μg/ml puromycin (Sigma-Aldrich) starting after 48 or 72 h.

### Colony-Picking or Fluorescence Activated Cell Sorting (FACS)

After 48 h of puromycin selection, emerging colonies were either manually picked or single cell sorted using a flow sorter (Sony Biotechnology Inc.). Manual picking of the colonies was carried out using a 10 μl pipette and Stereozoom®S4E light microscope (Leica microsystems). The individual colonies were identified under microscope and manually detached. Colonies were plated in a single well of a Matrigel® coated 96-well tissue culture plate (Sarstedt) containing E8 cell culture medium, supplemented with 10 μM ROCK inhibitor.

For cell sorting, Sony SH800 flow sorter was used to sort GFP positive (indicating successful entry of Cas9 plasmid) single cells, which were then plated on each well of the 96 well-plates containing E8 cell culture medium supplemented with 10 μM ROCK inhibitor. During both manual picking and cell sorting, medium was refreshed every 48 h (without ROCK inhibitor and puromycin) and the colonies were grown until they reached 70–80% confluency.

### PCR-Based Screening of All the Surviving Clones

Genomic DNA (gDNA) from all the surviving colonies originating from single colonies or cells, were isolated using Direct cell PCR lysis buffer (Viagen Biotech) supplemented with 20 μg/ml of Proteinase K (Thermo Fisher Technologies). The gDNA served as a template to identify cell lines with bi-allelic or mono-allelic deletion using a specific primer pair-based PCR screening with AmpliTaq gold DNA polymerase (Thermo Fisher Scientific). Conditions for screening PCR's are provided in Supplementary Material, primers used are listed in Supplementary Table 2.

### Targeted Sequencing of *MKRN3* KO Cell Lines

gDNA from WT and the *MKRN3* KO cell lines were PCR amplified with primers 200 bps upstream and downstream of *MKRN3* and the product was purified using A'SAP PCR clean up kit (Arcticzymes) according to the manufacturer's instructions. The purified PCR products were subjected to Next generation sequencing using Nextera DNA library preparation kit (Illumina Technologies) performed at the Institute for Molecular Medicine Finland (FIMM, Helsinki). The targeted sequencing data was analyzed with the Integrative Genomics Viewer (IGV) tool from Broad institute (27). The primers used for targeted sequencing of *MKRN3* are listed in Supplementary Table 2.

### Differentiation of hPSCs to *GNRH1*-Expressing Neurons

At >90% confluency, both WT and *MKRN3* KO hPSCs were differentiated into *GNRH1*-expressing neurons as described in Lund et al. (17). Briefly, the cells were treated with 2 μM Dorsomorphin (Selleckchem) and 10 μM SB431542 (Sigma-Aldrich) for 10 days to produce neuro-ectoderm, a further 10 days with 100 ng/ml FGF8 (Peprotech), and differentiation into post-mitotic neurons was stimulated by Notch inhibition using 20 μM DAPT (Sigma-Aldrich). The successful differentiation into *GNRH1*-expressing neurons was confirmed by morphological analysis, antibody staining to identify GnRH expressing cells and qPCR.

### RNA Isolation and Reverse Transcription

RNA extraction (Nucleospin RNA, Machery-Nagel) and cDNA synthesis by reverse transcription (iScript™ cDNA kit, BIO-RAD) were performed according to the manufacturer's instructions except that DNase treatment during RNA extraction was performed separately with RQ1 DNase (Promega), 1U/μg RNA, in presence of RNase inhibitor (Promega). The reverse transcription was performed in a regular thermal cycler with 1 μg of total RNA using iScript™ mix containing a blend of oligo (dT) and random hexamer primers. Conditions for RT PCR are provided in the Supplementary Material.

### Analysis of *GNRH1* Expression

The expression levels of *GNRH1* gene were measured by qPCR, normalized to Cyclophilin G (*PPIG*) and compared to expression in undifferentiated PSCs, as previously described (17). The normal range for *GNRH1* mRNA expression in WT cells was determined based on nine differentiation experiments (*n* = 9). Day 25, was used to characterize *GNRH1* expression in our protocol (17), and the same day was selected here for comparison of WT (*n* = 9) and *MKRN3* KO cell lines (*n* = 6) in all the experiments. Conditions for qPCR are provided in the Supplementary Material, the primers used in qPCR are listed in Supplementary Table 2.

### Immunocytochemistry

The cells were fixed using 4% paraformaldehyde for 15 min at room temperature (RT) and permeabilized for 7 min in 0.5% Triton X-100 (Sigma-Aldrich) diluted in PBS. Unspecific binding was blocked with UltraVision Protein Block (Thermo Fisher Scientific) for 10 min. Primary antibody incubation was performed overnight at 4°C and secondary antibody for 1 h at RT. Antibodies were diluted in PBS containing 0.1% Tween 20 (Sigma-Aldrich). Slides were mounted using VECTASHIELD® Mounting Medium with DAPI (Vector laboratories) for counterstaining the nuclei and microscopic images were obtained using ZEISS Axio Imager.Z2 upright epifluorescent microscope (Zeiss) using 10x/NA 0.3 and 20x/NA 0.8 EC PL APO CS2 objectives (Biomedicum Imaging Unit) and analyzed with ImageJ (NIH). All the antibodies and dilutions are listed in Supplementary Table 3.

### Generation of Stable Expression Cell Line and Cell Culture for Protein-Protein Interaction Studies in HEKs

For the generation of stable and inducible cell lines, gateway cloning (28) was employed to create *MKRN3* expression vector. *MKRN3* PCR-product with flanking site specific attachment sites (attB) and donor vector with site specific attachment sites (attP) were recombined by BP reaction to get the gateway compatible entry clone. Recombination between attB and attP sites results in attL and attR sites which are recombined by LR reaction between the entry clones and the MAC-C destination vector (20) to generate the MAC-tagged MKRN3 expression vector. Flp-In™ 293 T-REx cell lines (Invitrogen, Life Technologies, R78007) were co-transfected with the expression vector and the pOG44 vector (Invitrogen) using the DreamFect™ reagent (Oz Biosciences). Two days after transfection, culture medium was changed to DMEM/F12 (Thermo Fisher Scientific) supplemented with 10% FBS, 50 mg/mL penicillin, 50 mg/mL streptomycin (Sigma-Aldrich) and hygromycin B (Thermo Fisher Scientific) (100 μg/mL). The hygromycin B selection for isogenic clones was performed for 2 weeks.

The stable cell line was expanded to 30 × 150 mm plates. When cells reached 80% confluency, 1 μg/ml tetracycline (Sigma-Aldrich) was added for 24 h to express MAC-tagged MKRN3. For biotinylation labeling (BioID), 50 μM additional biotin (Thermo Fisher Scientific) was added at the same time with tetracycline. Cells deriving from 5 × 150 mm fully confluent dishes (approximately 5 × 10^7^ cells) were pelleted as one biological replicate. Three biological replicates for each condition and in total six biological replicates for two different conditions (with and without biotin) were collected. Samples were snap frozen at −80°C until further use.

### Affinity Purification of Protein Complexes and Mass Spectrometry (MS)

The cells with only tetracycline induction were used for affinity purification (29). The cells treated with tetracycline and biotin were applied for BioID purification as previously described (20). Purified protein complexes (AP-MS)/proteins (BioID) were processed and digested to peptides for MS analysis. The analysis was performed on Qrbitrap Elite hybrid mass spectrometer using Xcalibur version 2.0.7 SP1 (Thermo Scientific) coupled with an EASY nLC 1000-reverse phase HPLC system via an electrospray ionization sprayer (Thermo Fisher Scientific). MS was performed in data-dependent acquisition mode using Fourier transform mass spectrometry full scan (300–1,700 m/z) resolution of 60,000 and collision-induced dissociation scan of top 20 most abundant ions.

### Data Processing

For protein identification, Thermo.RAW files were searched against reviewed selected human UniProtKB/SwissProt database (http://www.uniprot.org/, version 2017-02) with SEQUEST search engine. The decoy database was the reverse of the target database. All data were reported based on 95% confidence for protein identification, as determined by the false discovery rate (FDR) ≤5%. Contaminant Repository for Affinity Purification (CRAPome, http://www.crapome.org/) (30) database and in-house GFP samples database were used as controls with a cut-off frequency of 20% (411 runs from CRAPome database and 100 runs of in house GFP control samples) for identification of high-confidence interactions (HCIs). HCIs data were imported into Cytoscape 3.4.0 to construct protein interaction network. The known prey-prey interaction data were obtained from IMEx database (http://www.imexconsortium.org/) (31). Gene ontology classification analysis was based on DAVID bioinformatics resource (https://david.ncifcrf.gov/) (32). We also compared the HCI partners of MKRN3 with proteins encoded by 371 genes, that are associated with puberty timing according to large population based studies (33, 34). In addition, we investigated the HCIs to proteins encoded by 37 genes implicated in congenital hypogonadotropic hypogonadism (CHH) (35–40).

### Statistics

The relative *GNRH1* expression in WT H9 and MKRN3 KO cell lines Del 1 and Del 2 was tested with unpaired *t*-test after logarithmic transformation to normalize the distributions. *P*-value < 0.05 was accepted to indicate statistical significance.

## Results

### CRISPR/Cas9 Based Generation of *MKRN3* KO Cell Lines in hPSCs

**Figure 1:**
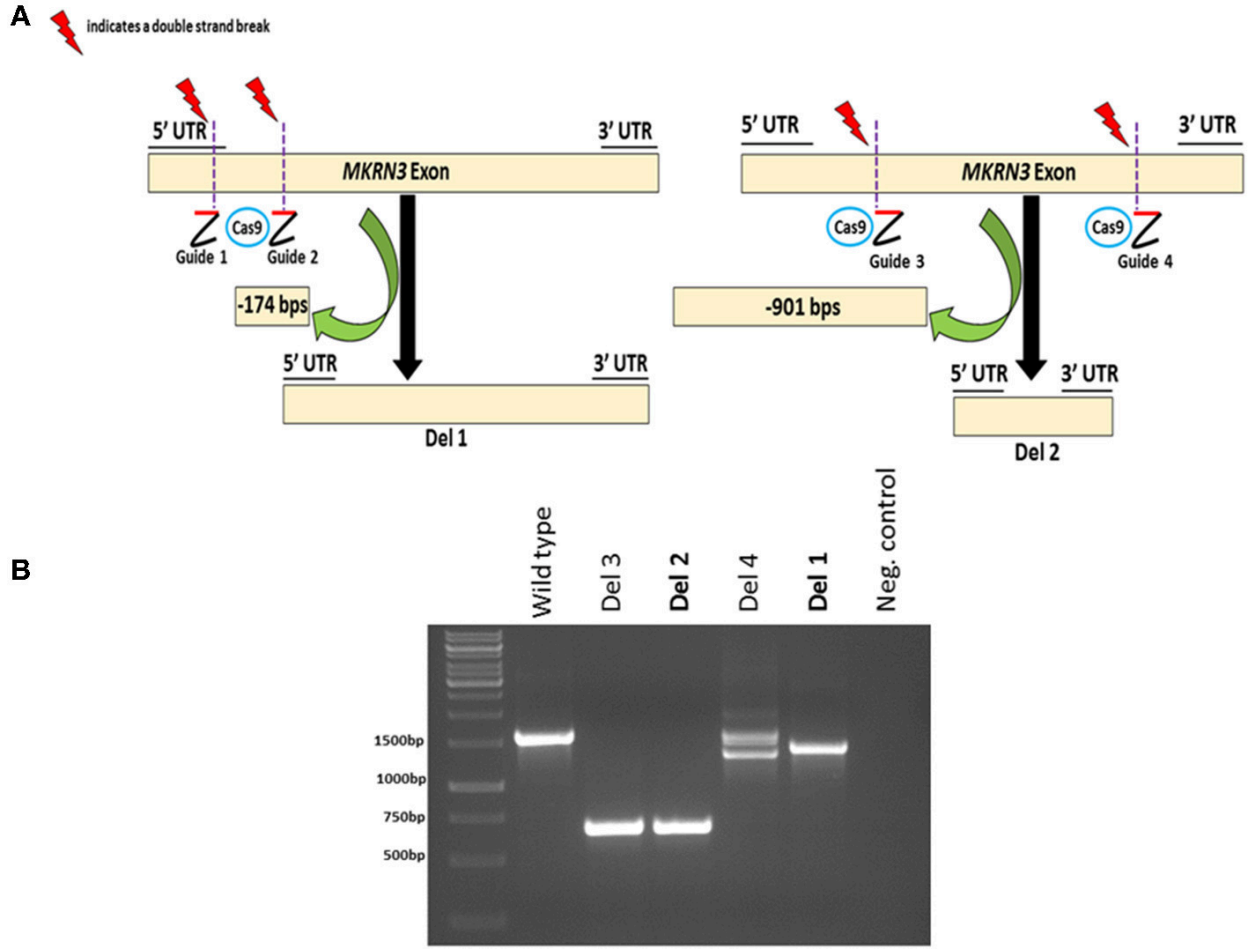
CRISPR/Cas9-based generation of two bi-allelic MKRN3 KO cell lines (Del 1 and Del 2) in human pluripotent stem cell line H9 and verification of CRISPR/Cas9 induced deletions at genomic DNA level. **(A)** Left panel, Del 1 has a deletion of 174 bps, translating to a deletion of 51 amino acids (11% of protein) including the translation start site. Right panel, Del 2 has a deletion of 901 bps, translating to 300 amino acid deletion (60% of protein). **(B)** The presence or absence of CRISPR/Cas9-induced deletions in *MKRN3* was verified by PCR amplification of the region of interest from genomic DNA of WT human pluripotent H9 cells, and in CRISPR/Cas9 edited cells. Four examples of edited cell lines are shown (Del 1–Del 4). The same pair of guide RNAs, designed to delete ~900 bps, was used for editing Del 2 and Del 3 cell lines, whereas two different gRNA pairs were used to edit Del 1 and Del 4 cell lines. The PCR products were visualized on a 1.5% agarose gel.

**Figure 2:**
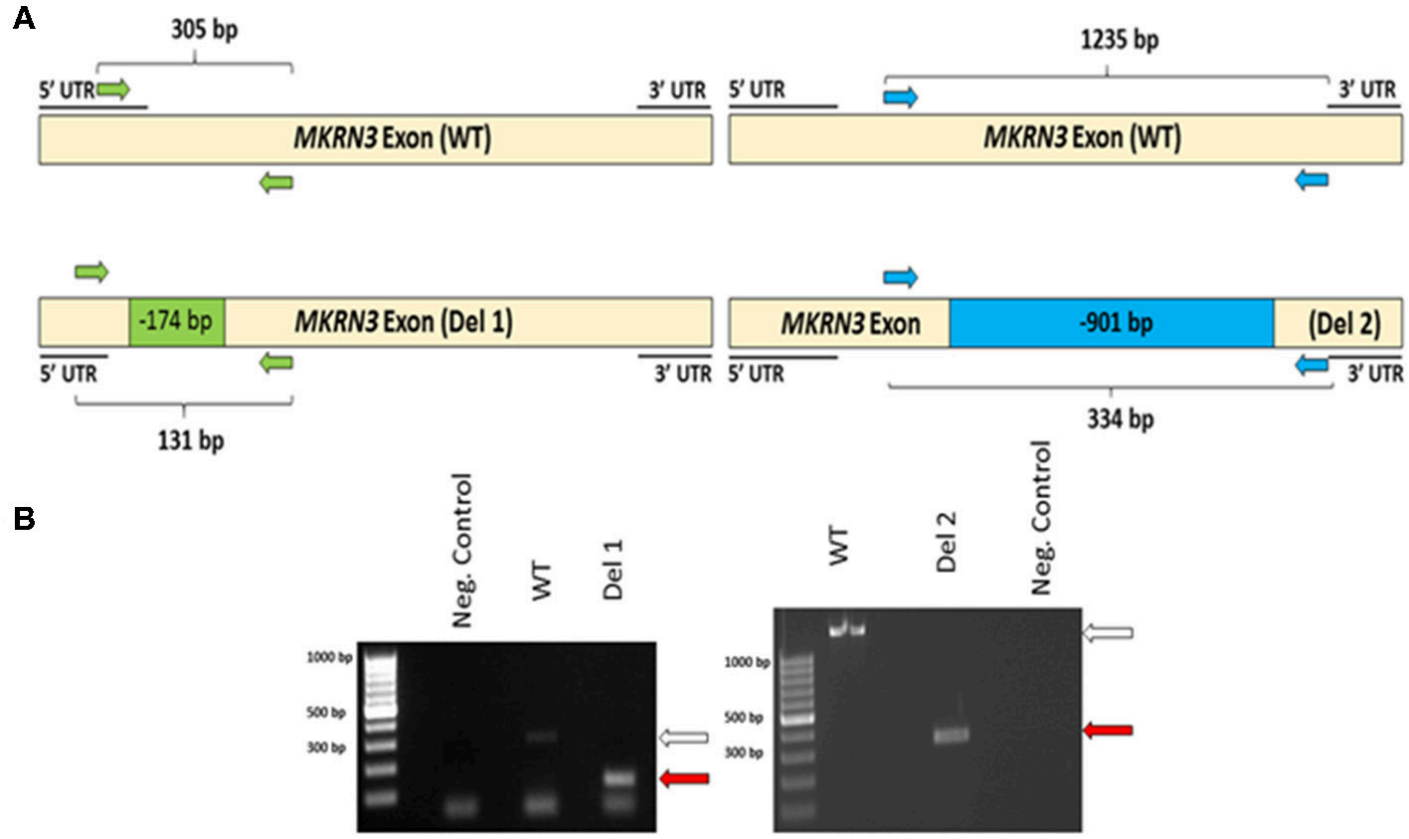
**(A)** Schematic of *MKRN3* cDNA and the two pairs of primers (green and blue arrows) employed in detection of WT, Del 1, (left panel) and Del 2, (right panel). cell lines. In WT cells, the expected product sizes are 305 and 1,235 bp, in Del 1 cells 131 bp and in Del 2 cells 334 bp. **(B)** *MKRN3* amplification from the cDNA of the two bi-allelic *MKRN3* deletion cell lines Del 1, (left panel) and Del 2, (right panel). White arrow indicates the expected wild type (WT) product sizes (305 and 1,235 bp, respectively) and the red arrows indicate the expected sizes (131 and 334 bp, respectively) for the PCR products from Del 1 and Del 2 cell lines. The cDNA used is from day 25 of the protocol generating *GNRH1*-expressing neurons. The PCR products were visualized on a 1.5% agarose gel.

To investigate the impact of MKRN3 deficiency in the differentiation of *GNRH1*-expressing neurons, we generated two hPSC lines with bi-allelic deletions of *MKRN3* using CRISPR/Cas9. Bi-allelic KO cell lines were generated, as heterozygous *MKRN3* mutations cause CPP only when inherited from the father, and we could not selectively target the desired deletion to the paternal allele. The schematic indicates the guide targeting areas and deletions in these cell lines (Figure 1A). In brief, two pairs of RNA guides spanning different lengths on the only exon of *MKRN3* together with Cas9 resulted in deletions of either −174 or −901 bps. For further studies, we chose two apparently homozygous KO lines Del 1 (small deletion, −174 bps), and Del 2 (large deletion, −901 bp), which were validated to carry desired deletions by PCR (Figure 1B) and targeted sequencing Supplementary Figures 1, 2). The absence of functional *MKRN3* expression was further validated by performing PCR with cDNAs from the WT and *MKRN3* KO cell lines as template and with primers flanking the deletion regions (Figure 2A). The length of the transcripts produced from the KO clones corresponded to the size of deletions seen at DNA level (Figure 2B), consistent with the bi-allelic deletion in the coding region of *MKRN3*.

### Differentiation of *MKRN3* KO and WT hPSCs to *GNRH1*- Expressing Neurons

**Figure 3:**
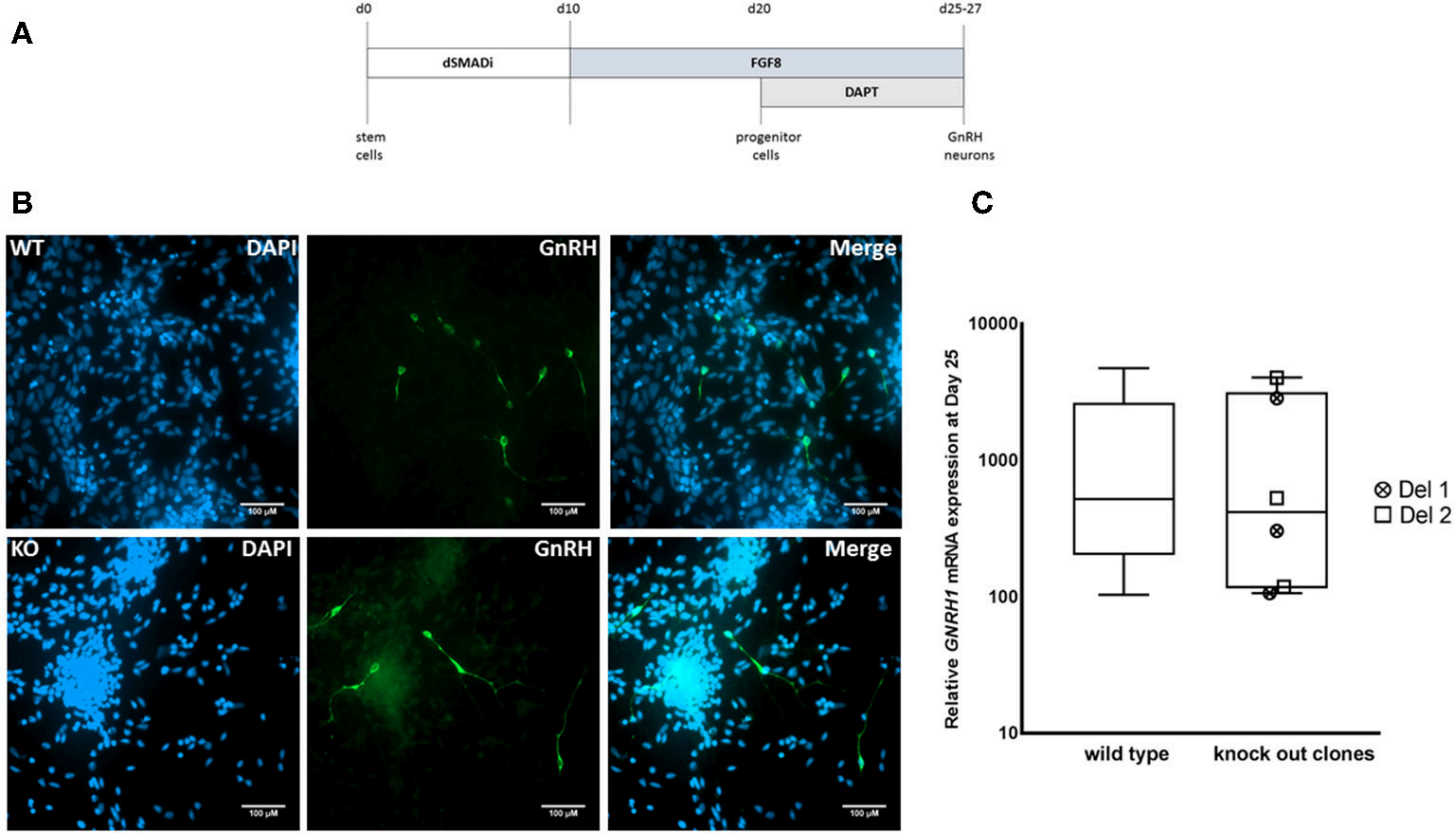
**(A)** Schematic for the generation of *GNRH1*-expressing neurons from hPSCs (17). In brief, dual SMAD inhibition by Dorsomorphin and SB431542 was applied for the first 10 days, followed by 10 days of FGF8 treatment and final stage of neuronal maturation for 5–7 days by combining FGF8 with Notch inhibition by DAPT. This differentiation methodology has been adapted from a previously published protocol and the figure has been modified from the original publication by Lund et al. (17). **(B)** Immunocytochemistry in WT and Del 2 derived day 25 GnRH-positive neurons (green) nuclear stained with DAPI (blue), scale bars represent 100 μM. **(C)** The relative expression of *GNRH1* in WT H9 cells (*n* = 9) and in two bi-allelic *MKRN3* KO cell lines (Del 1 and Del 2) following three independent repeats (*n* = 6) of differentiation into *GNRH1*-expressing neurons (17). Expression levels are relative to day 0 hPSCs.

We next differentiated *MKRN3* KO cell lines Del 1 and Del 2 into *GNRH1*-expressing neurons, according to our recently described protocol (Figure 3A) (17). Both cell lines could be differentiated into neuronal progenitors and further into *GNRH1*-expressing neurons (Figure 3B). At day 25 of differentiation the relative *GNRH1* mRNA expression between the *MKRN3* KO cell lines and WT H9 cells (Figure 3C), did not differ (*P* = NS). The mRNA expression levels of *OTX2*, a transcriptional regulator of *GNRH1* (41), together with *OTX1*, both of which are enriched in GnRH-neurons of mice (42) were not significantly different between WT and Del 2 (*n* = 3) cells at day 25 of the differentiation protocol (Supplementary Figure 3).

### MKRN3 and Its Protein Interaction Partners

**Figure 4:**
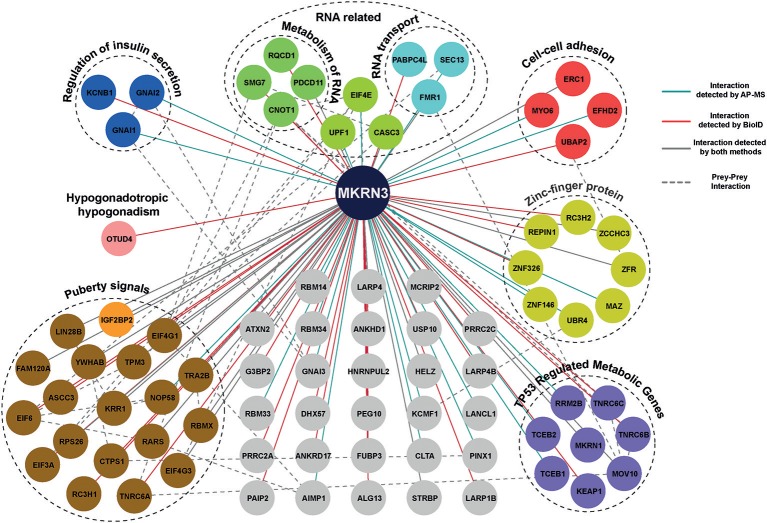
Protein-protein interaction map of MKRN3. The network represents PPIs of MKRN3 detected by different purification methods. Blue edges represent experimentally validated interaction by AP-MS; Red lines represent interactions detected by BioID purification; Overlap of two purification methods is shown with gray color, while a dashed line describes prey-prey interactions. The colored nodes represent the different clusters of prey proteins.

Using MS analysis, we identified 104 HCIs including both transient and stable interactions for MKRN3 (Supplementary Table 4). These 104 HCIs total for 81 unique HCIs (Figure 4), were classified into functional groups according to their annotation categories involved in various pathways regulating cellular processes, mainly the regulation of insulin secretion, cell-cell adhesion, TP53-regulated cell metabolism, transcriptional repression and RNA activity related interactors (32). Importantly, our MS data revealed that MKRN3 physically interacts with as many as 19 proteins (including LIN28B) encoded by genes, which have been previously associated with the age at menarche (33, 34). In addition, MS data identified OTUD4, a protein implicated in CHH (43), as HCI partner for MKRN3.

## Discussion

In mice, expression of *Mkrn3* decreases during postnatal development, and in humans, circulating MKRN3 levels decrease toward the onset of puberty in both sexes (44, 45). However, investigation of peripheral MKRN3 levels does not allow for mechanistic explanation of the role of MKRN3 in regulation of puberty in humans. Therefore, we employed our recently described differentiation protocol generating *GNRH1-*expressing neurons (17) and genetically engineered hPSCs to investigate if *MKRN3* is required for GnRH neuron differentiation in this model. In addition, we investigated PPI partners of MKRN3 in HEK cells and paid special attention to possible interactors encoded by puberty-related genes.

Although several studies have reported that paternally inherited mutations in *MKRN3* cause CPP (46), the underlying mechanisms still remain to be explained (1, 2). In our study, we first examined the role of *MKRN3* on *GNRH1* expression by using two bi-allelic *MKRN3* KO clonal cell lines (Del 1 and Del 2), generated with CRISPR/Cas9 technology. These cell lines were subsequently differentiated into *GNRH1*-expressing neurons through our recently described differentiation protocol, which is based on dual SMAD inhibition, FGF8 treatment and Notch inhibition (17). Our results show that both the Del 1 and Del 2 cell lines could be successfully differentiated into *GNRH1*-expressing neurons, suggesting that *MKRN3* is dispensable for the differentiation. Intriguingly, *GNRH1* expression of Del 1 and Del 2 cell lines did not differ from the controls, which suggests that *MKRN3* does not directly alter *GNRH1* expression. However, there are many environmental and metabolic signals postnatally, that may regulate further maturation and function of GnRH neurons (47, 48). While *MKRN3* does not affect the GnRH neuron differentiation or their ability to express *GNRH1* in our model, there are other potential mechanisms by which *MKRN3* may regulate GnRH neuron function. For example, major regulation of GnRH neuron activity is carried out by Kisspeptin and KNDy neurons in the hypothalamus, and putative role of MKRN3 on these neurons is not known. The possibility of other MKRNs compensating for the loss of MKRN3 in Del 1 and Del 2 cell lines cannot be ruled out. On the other hand, hPSC-based disease modeling is the closest available approach to model human neuronal disorders (49), and with regard to MKRN3, murine models have not yet been reported. What is known so far, however, is that *MKRN3* is not significantly enriched or depleted in mouse GnRH neurons (42).

Given that we could not find a mechanistic cue for the role of MKRN3 in *GNRH1* expression in our stem cell model, we considered alternative approaches to gain insight on how this putative E3-ligase might affect puberty timing. In brief the method investigating PPIs employs a MAC-tag system that contains affinity tags for affinity purification-mass spectrometry (AP-MS) as well as the biotin ligase BirA^*^ for BioID. Therefore, a single construct is sufficient to identify both stable and transient interactions (20). We did not carry out PPI studies in our stem cell model, since the efficient cloning of all different tagged MKRN3 constructs to hPSCs was beyond the scope of this work. While this is a potential caveat, it is important to note that HEK cells, one of the most widely employed human cell lines in biomedical research (50), are not unsuitable for our purpose since they exhibit some neuronal properties (23). In addition, our laboratory is extremely experienced in using HEK cell line-based platform, which allows rapid and efficient generation of expression constructs and subsequent data analysis. Based on the list of HCI proteins for MKRN3, we found that MKRN3 might be involved in cell-cell adhesion, RNA metabolism and insulin secretion. Insulin has been suggested to modify GnRH neuron function in humans, and insulin sensitivity is thought to be an important factor in the initiation of puberty (1, 51, 52). MKRN3 interacts with a group of proteins encoded by metabolic genes, which in turn, are regulated by TP53, a tumor suppressor gene involved in several cancers and implicated in puberty (53). Other PPI partners of MKRN3 are involved in RNA transport and metabolism, which remains less explored areas in terms of puberty.

In addition, we found that MKRN3 interacts with 20 proteins encoded by puberty timing-associated genes of which *LIN28B* is one of the most established puberty-related genes in genetic studies (54, 55) and *OTUD4* represents the only puberty-related disease gene (43). Although *LIN28B* has been associated with the age at menarche, adult height and childhood growth (56–60), and is known to be a negative regulator of let-7 class of microRNAs (61), the mechanism by which it regulates puberty timing is unknown (1, 62, 63). In rats and non-human primates *Lin28b* expression declines in the hypothalamus at puberty (64), and in mice, expression of both *Lin28b* and *Mkrn3* is reduced prior to puberty (7, 65). Our findings provide evidence of physical interaction between human LIN28B and MKRN3 and it is tempting to speculate that they may act in concert to regulate the timing of puberty. We also examined MKRN3 interactions among 37 genes implicated in CHH (35–40), and found that MKRN3 interacts with OTUD4. *OTUD4* is a deubiquitinase coding gene (43), and it is intriguing to hypothesize that it may regulate the timing of puberty by counteracting the effects of MKRN3. Further studies are required to confirm this hypothesis. Finally, MKRN3 interacts with other zinc finger genes (ZNFs) which are transcriptional repressors and have an important role during puberty (66), however, we could not observe MKRN3's interaction with ZNFs critical for puberty onset, such as ZNF573 and GATAD1 (66). Even so, the interactions observed invite the possibility of *MKRN3* being an epigenetic transcriptional repressor involved in puberty timing.

In conclusion, our results suggest that *MKRN3* is dispensable for the GnRH neuron differentiation process and *GNRH1* expression during differentiation from human pluripotent stem cells. Although the mechanism by which *MKRN3* regulates humans sexual maturation remains unclear, our results suggest that MKRN3 may act in concert with previously identified puberty-related proteins, and it is enticing to hypothesize that this interaction is translated to functional cooperation in terms of puberty timing. Further studies are required before we understand how and why the HPG axis activates prematurely in children with a paternally inherited loss-of-function mutations in *MKRN3*.

## Author Contributions

VY, SV, TT, and TR planned the project. TT, MV, and TR supervised the project. VY, CL, KP, and KL carried out the stem cell work. XL and MV designed the protein interaction study. XL and JK carried out the protein interaction work. VY wrote the manuscript and TR edited the manuscript.

### Conflict of Interest Statement

The authors declare that the research was conducted in the absence of any commercial or financial relationships that could be construed as a potential conflict of interest.

## Abbreviations

CPP: central precocious puberty
MKRN3: makorin ring finger protein 3
hPSCs: human pluripotent stem cells
WT: wild type
KO: knock-out
CRISPR: clustered regularly interspaced palindromic repeats
Cas9: CRISPR associated protein 9
bps: base pairs
gRNA: guide RNA DNA template
GFP: green fluorescent protein
PPI: protein-protein interaction
HCI: high-confident interactions
CHH: congenital hypogonadotropic hypogonadism.
