# Supplementary material for: MKRN3 Interacts With Several Proteins Implicated in Puberty Timing but Does Not Influence GNRH1 Expression
Source: Front Endocrinol (Lausanne). 2019 Feb 8;10:48. doi: 10.3389/fendo.2019.00048 (PMC6375840; doi:10.3389/fendo.2019.00048)
Supplement: Supplementary file 1 [file Data_Sheet_1.docx]

**Supplementary material**

**MKRN3 interacts with proteins implicated in puberty timing including OTUD4 and LIN28B and does not directly influence *GNRH1* expression**

Venkatram Yellapragada, Xiaonan Liu, Carina Lund, Johanna Känsäkoski, Kristiina Pulli, Sanna Vuoristo, Karolina Lundin, Timo Tuuri, Markku Varjosalo*, Taneli Raivio*

*Authors contributed equally

Correspondence: [taneli.raivio@helsinki.fi](mailto:taneli.raivio@helsinki.fi)

1. **Supplementary data**
   1. **Guide RNA production (gRNA-PCR)**

The gRNA PCR components used are 1 ng of U6 promoter, 1 ng of U6 terminator, 50 pmol of forward and reverse primer together with 2 pmol guide oligo in a 100 µl reaction. The conditions for PCR program are 35 cycles of 98°C for 10 s, 52°C for 30 s and 72°C for 12 s. The PCR amplified guides were then purified and concentration was measured before transfection.

- 1. **MKRN3 KO Screening PCRs**

All the screening PCRs were performed using Amplitaq gold DNA polymerase and a regular PCR program consisting 40 cycles of 98°C for 1 min, 60°C for 1 min and 72°C for 1 min.

- 1. **Real-time quantitative PCR (qPCR)**

Real-time quantitative PCR included 25ng of first-strand cDNA, HOT FIREPol® EvaGreen® qPCR Mix Plus (Solis BioDyne), and 0.5 µM forward and reverse primers. Reactions were performed in a LightCycler® 480 (Roche) for 45-50 cycles of 95°C for 15 s, 60°C for 20 s and 72°C for 20 s.

- 1. **RT-PCR**

RT PCRs were performed as per the manufacturer’s instructions (BIO-RAD iScript™) and 5 min at 25°C, 20 min at 46°C and 1 min at 95°C as PCR conditions

1. **Supplementary Figures and Tables**

**2.1 Supplementary Figures**

**2.1.1 Supplementary Figure
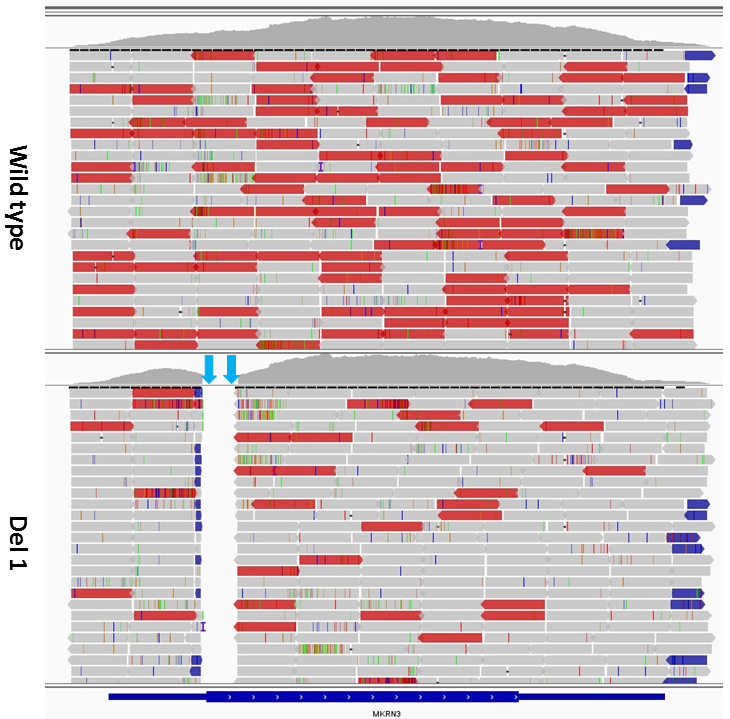
1.**

An alignment image from the IGV software. WT *MKRN3* gene is shown at the bottom (blue bar), alignment of targeted sequence reads indicates the presence of bi-allelic deletion of *MKRN3* (indicated by blue block arrows) in Del 1 *MKRN3* KO human pluripotent stem cell line.

**
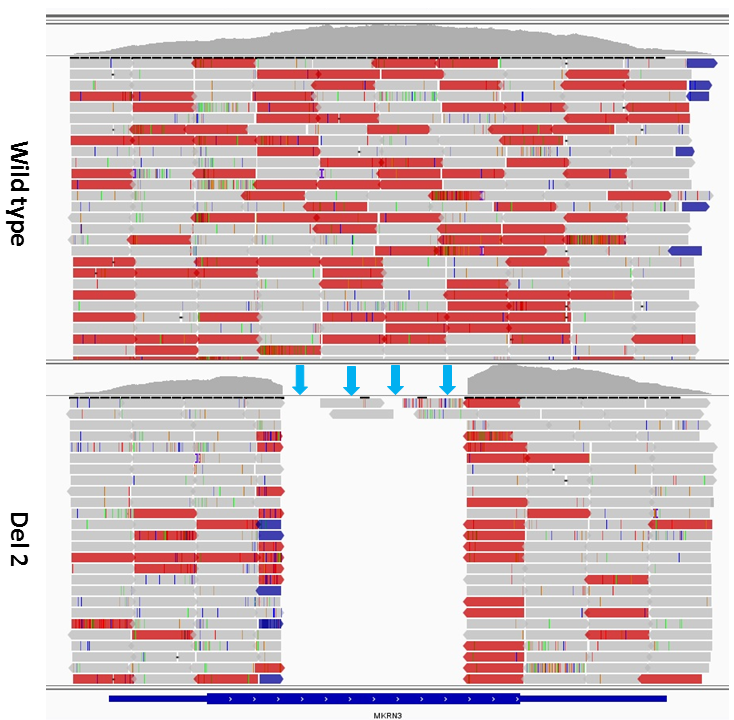
2.1.2 Supplementary Figure 2.**

An alignment image from the IGV software. WT *MKRN3* gene is shown at the bottom (blue bar), alignment of targeted sequence reads indicates the presence of bi-allelic deletion of *MKRN3* (indicated by blue block arrows) in Del 2 *MKRN3* KO human pluripotent stem cell line.

**2.1.3 Supplementary Figure 3.**

*
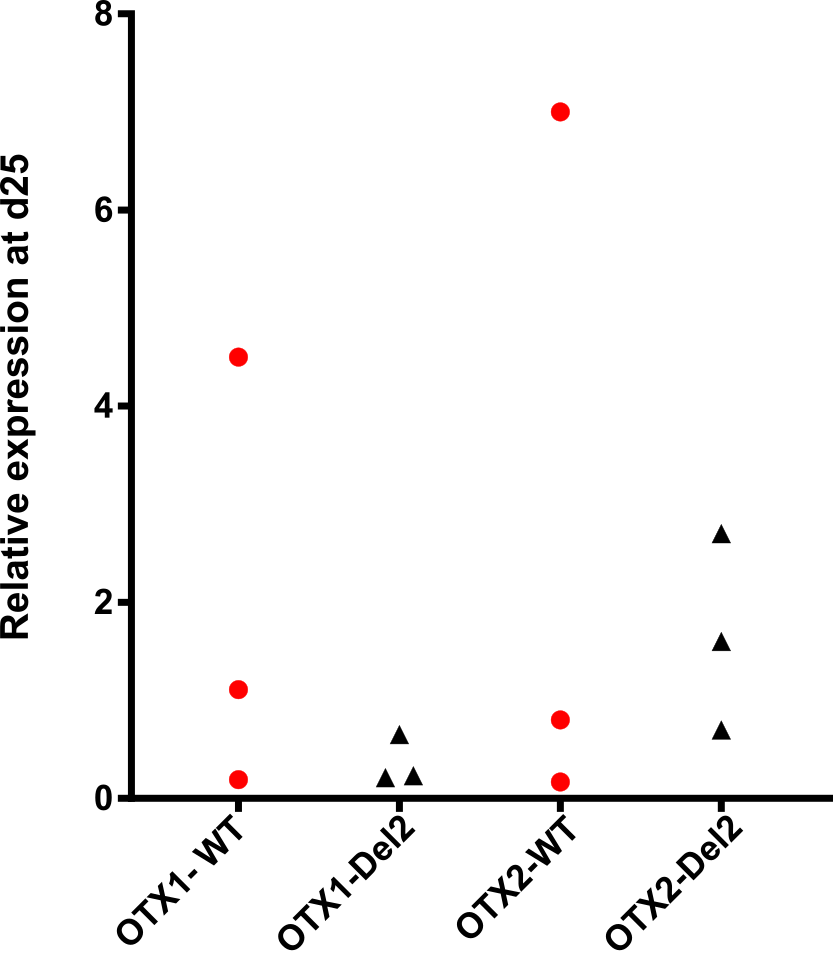
*

Relative *OTX1* and *OTX2* expression levels from the WT (n=3) and *MKRN3* KO (Del 2) (n=3) cells at day 25 of GnRH differentiation protocol.

**2.2 Supplemental Tables**

**2.2.1 Supplementary table 1**

CRISPR/Cas9 related RNA oligos, U6 components and primers

**2.2.2 Supplementary table 2**

All the primers used for targeted sequencing, KO screening and qPCRs

**2.2.3 Supplementary table 3**

Antibodies used in the study

**2.2.4 Supplementary table 4**

Complete list of MKRN3 HCIs

All the four supplementary tables are appended in the supplementary tables excel sheet.
